# The Adaptor Molecule Nck Localizes the WAVE Complex to Promote Actin Polymerization during CEACAM3-Mediated Phagocytosis of Bacteria

**DOI:** 10.1371/journal.pone.0032808

**Published:** 2012-03-20

**Authors:** Stefan Pils, Kathrin Kopp, Lisa Peterson, Julia Delgado Tascón, Naja J. Nyffenegger-Jann, Christof R. Hauck

**Affiliations:** 1 Lehrstuhl Zellbiologie, Universität Konstanz, Konstanz, Germany; 2 Zentrum für Infektionsforschung, Universität Würzburg, Würzburg, Germany; 3 Konstanz Research School Chemical Biology, Universität Konstanz, Konstanz, Germany; University of Bonn, Germany

## Abstract

**Background:**

CEACAM3 is a granulocyte receptor mediating the opsonin-independent recognition and phagocytosis of human-restricted CEACAM-binding bacteria. CEACAM3 function depends on an intracellular immunoreceptor tyrosine-based activation motif (ITAM)-like sequence that is tyrosine phosphorylated by Src family kinases upon receptor engagement. The phosphorylated ITAM-like sequence triggers GTP-loading of Rac by directly associating with the guanine nucleotide exchange factor (GEF) Vav. Rac stimulation in turn is critical for actin cytoskeleton rearrangements that generate lamellipodial protrusions and lead to bacterial uptake.

**Principal Findings:**

In our present study we provide biochemical and microscopic evidence that the adaptor proteins Nck1 and Nck2, but not CrkL, Grb2 or SLP-76, bind to tyrosine phosphorylated CEACAM3. The association is phosphorylation-dependent and requires the Nck SH2 domain. Overexpression of the isolated Nck1 SH2 domain, RNAi-mediated knock-down of Nck1, or genetic deletion of Nck1 and Nck2 interfere with CEACAM3-mediated bacterial internalization and with the formation of lamellipodial protrusions. Nck is constitutively associated with WAVE2 and directs the actin nucleation promoting WAVE complex to tyrosine phosphorylated CEACAM3. In turn, dominant-negative WAVE2 as well as shRNA-mediated knock-down of WAVE2 or the WAVE-complex component Nap1 reduce internalization of bacteria.

**Conclusions:**

Our results provide novel mechanistic insight into CEACAM3-initiated phagocytosis. We suggest that the CEACAM3 ITAM-like sequence is optimized to co-ordinate a minimal set of cellular factors needed to efficiently trigger actin-based lamellipodial protrusions and rapid pathogen engulfment.

## Introduction

Several human-restricted pathogens target surface receptors of the carcinoembryonic antigen-related cell adhesion molecule (CEACAM) family to contact their host [1]. In particular, CEACAM1, CEACAM3, CEA (the product of the *CEACAM5* gene), and CEACAM6 can serve as microbial receptors. As CEACAM-recognition has evolved independently in multiple Gram-negative bacteria including *Haemophilus influenzae*, *Moraxella catarrhalis*, *Neisseria gonorrhoeae* and *N. meningitidis*, as well as uropathogenic *Escherichia coli*, this trait seems to confer a selective advantage to these microbes in vivo [2]. All CEACAM-binding bacteria colonize the mucosal surface of the human nasopharynx, intestine or urogenital tract, where CEACAM family members are expressed on the apical membrane of polarized epithelial cells [3]. In vivo experiments employing a humanized mouse model have demonstrated that CEACAM recognition can strongly promote the initial establishment of microorganisms on the mucosa [4]. In this context, bacterial stimulation of CEACAMs enhances the adhesiveness of the infected host cells, thereby suppressing the exfoliation of the superficial epithelial layer [4], [5].

On the other hand, CEACAM family members such as CEACAM1, CEACAM3, or CEACAM6 are found on various hematopoietic cells. A common splice variant of CEACAM1, which is present on mature B-cells and CD4-positive T-cells, contains two immunoreceptor tyrosine-based inhibition motifs (ITIMs) and has been reported to interfere with antibody production by B-cells [6], [7] or CD4 T-cell proliferation, respectively, upon stimulation by CEACAM-binding bacteria [8]. Though this report has been challenged by recent observations that both CEACAM-binding and non-binding *N. gonorrhoeae* stimulate T-cell proliferation and cytokine secretion to the same extent [9], engagement of CEACAM1 on immune cells by pathogenic microbes might interfere with some effector functions of hematopoietic cells and thereby provide a selective advantage [10]. Interestingly, human granulocytes express a peculiar member of the CEACAM family, CEACAM3, which shares a high degree of homology in its extracellular, bacteria-binding part with CEACAM1, CEA, and CEACAM6 [11]. In contrast to CEACAM1, the CEACAM3 cytoplasmic domain encompasses a tyrosine-based sequence that is reminiscent of an immunoreceptor tyrosine-based activation motif (ITAM). Canonical ITAM sequences are found in the cytoplasmic parts of T-cell and B-cell receptor as well as Fc gamma receptor (FcγR) subunits [12]. ITAMs are critical for transducing stimulatory signals and, in the case of phagocytes, promote bactericidal activities of these effector cells [13]. Interestingly, engagement of CEACAM3 by bacteria has been shown to result in rapid phagocytosis and elimination of the bacteria in an acid intracellular compartment [14], [15]. Therefore, CEACAM3-mediated recognition and opsonin-independent phagocytosis might be a specific adaptation of the human innate immune system to balance the colonization of mucosal surfaces by CEACAM-binding microbes. In line with the idea that CEACAM3-mediated engulfment by granulocytes is to the disadvantage of the recognized microbes, several studies have demonstrated that this uptake process is mechanistically distinct from CEACAM1, CEACAM6 or CEA-mediated internalisation [16]–[19]. For example, CEACAM3-mediated internalisation strictly depends on actin cytoskeleton dynamics and does not involve cholesterol- and sphingolipid-rich membrane microdomains [18], [19]. Moreover, whereas CEA and CEACAM6 are glycosylphosphatidyl-inositol-anchored proteins and CEACAM1 lacking the cytoplasmic domain is competent for bacterial uptake, cytoskeletal rearrangements and efficient phagocytosis via CEACAM3 depend on the integrity of the cytoplasmic ITAM-like sequence [15], [16], [19], [20]. Starting with receptor clustering by multivalent bacteria, several steps in the CEACAM3-initiated signalling pathway have been delineated in primary granulocytes and CEACAM3-transfected cell lines. Most importantly, tyrosine phosphorylation of the ITAM-like sequence by Src-family protein tyrosine kinases (PTKs) appears as the initial event, which guides the assembly of a transient signalling complex and which is blocked by pharmacological inhibitors of Src family kinases [17], [18], [21]. Upon phosphorylation of tyrosine residue Y230 within the ITAM-like sequence, this phospho-tyrosine serves as a docking site for the guanine nucleotide exchange factor (GEF) Vav [22]. Direct association of the CEACAM3 ITAM with the SH2 domain of Vav provides a short-cut between receptor engagement and GTP-loading of the small G-protein Rac, which is a critical regulator of actin polymerization. Accordingly, dominant-negative versions of Rac, but not the closely related G-protein Cdc42, severely reduce opsonin-independent phagocytosis of CEACAM-binding bacteria by primary human granulocytes [15]. In line with a central role of Rac in this process, a rapid increase in GTP-loaded Rac as well as the formation of large lamellipodial protrusions is observed in granulocytes infected with CEACAM-binding gonococci [15], [23]. However, which Rac-dependent effectors contribute to actin polymerization in the vicinity of CEACAM3-bound bacteria and how these effectors are recruited to the sites of bacterial uptake is currently unknown.

In this study, we identified the adaptor molecules Nck1 and Nck2 as novel interacting partners of CEACAM3. Biochemical analyses demonstrated that Nck1 and Nck2, but not other adapter molecules such as Grb2, CrkL, or SLP-76, bound to the ITAM-like sequence of CEACAM3 in a phosphorylation-dependent manner. In microscopic investigations, Nck1 clustered together with the receptor at sites of bacterial uptake and overexpression of the isolated SH2-domain of Nck1, siRNA-mediated knock-down of Nck1, or genetic deletion of Nck1 and Nck2 interfered with CEACAM3-mediated phagocytosis and the generation of lamellipodial protrusions. Interestingly, Nck was found in a CEACAM3-containing complex together with the Rac effector WAVE2, which promoted f-actin polymerization during uptake of CEACAM3-associated bacteria. Interference with WAVE complex function by RNAi or overexpression of dominant-negative WAVE2 reduced CEACAM3-mediated internalization. These results suggest that CEACAM3-associated Nck helps to bring together Rac effector molecules such as WAVE with receptor-bound Rac activators such as the GEF Vav to promote the local actin cytoskeleton rearrangements necessary for the opsonin-independent internalisation of CEACAM-binding bacteria.

## Materials and Methods

### Bacteria

*Neisseria gonorrhoeae* strain MS11 lacking pili and constitutively expressing Opa_CEA_ (Opa_52_; strain N309) was kindly provided by Thomas Meyer (Max-Planck-Institut für Infektionsbiologie, Berlin, Germany). Gonococci were cultured as described previously [15]. For gentamicin protection assays, overnight grown bacteria were taken from GC-agar plates supplemented with vitamins. For microscopy or uptake assays by flow cytometry, bacteria were labelled as indicated with either 0.2 µg/ml Pacific Blue-succinimidylester, Carboxyfluorescein-succinimidylester (CFSE), Rhodamine-Red-succinimidylester, or AlexaFluor647- succinimidylester (Invitrogen, Carlsbad, CA) as described [22].

### Cell culture

Human embryonic kidney epithelial 293T cells (293 cells; ACC-635, German collection of microorganisms and cell cultures, DSMZ, Braunschweig, Germany) were cultured in DMEM supplemented with 10% calf serum (CS). HeLa cells stably expressing CEACAM3 [22] were provided by W. Zimmermann (Tumor Immunology Laboratory, LMU München, Germany) and cultured in DMEM supplemented with 10% fetal calf serum (FCS). Nck1 and Nck2-deficient mouse fibroblasts (Nck1−/−Nck2−/−cells) and the heterozygous control cells (Nck1+/−Nck2+/−cells) were described previously [24] and were provided by T. Pawson (Samuel Lunenfeld Research Institute, Mount Sinai Hospital, Toronto, Canada). Fibroblasts were cultured in DMEM supplemented with 10% fetal calf serum (FCS) and non-essential amino acids on gelatine-coated culture dishes. All cells were subcultured every 2–3 days. Cells were counted and assayed for viability (>90%) with a Casy Cell Counter (Innovatis, Bielefeld, Germany). 2.5×10^5^ cells were seeded into each well of a gelatine-coated 48 well plate for gentamicin protection assays. For confocal laser scanning microscopy, 7.5×10^4^ cells were seeded in 24 well plates on glass cover slides coated with a mixture of fibronectin (4 µg/ml) and poly-L-lysine (10 µg/ml) in PBS.

### Recombinant DNA

Plasmids encoding CEACAM3-HA wildtype (WT) or the CEACAM3 mutant lacking the complete cytoplasmic domain (CEACAM3 ΔCT) and CEACAM3 WT-RFP were described previously [15], [22]. The HA-tagged CEACAM3 WT or CEACAM3 ΔCT were amplified using primers CEACAM3-IF-sense 5′-GAAGTTATCAGTCGATACCATGGGGCCCCCCTCAGCC-3′ and CEACAM3-IF-antisense 5′-ATGGTCTAGAAAGCTTGCAGCGTAATCTGGAACGTCATATGG-3′ and cloned with the InFusion cloning kit into pDNR-dual (Clontech, Mountain View, CA). CEACAM3 variants with an additional carboxy-terminal green-fluorescent protein (GFP), mCerulean, or mKate tag were generated by Cre/lox recombination from pDNR-dual into pLPS3'EGFP, pLPS3'mCerulean, or pLPS3'mKate as described [21]. pLPS3'mCerulean-loxp and pLPS3'mKate-loxp were constructed by replacement of the EGFP coding sequence in pLPS3'EGFP (Clontech, Mountain View, CA). mCerulean cDNA (kindly provided by D. Piston, Department of Molecular Physiology and Biophysics, Vanderbilt University, TN) was amplified using primers 5′-ACTACCGGTCGTGGTGAGCAAGGGCGAG-3′ and 5′-ACTGCGGCCGCTTATTTGTACAGTTCGTCC-3′, whereas mKate cDNA (kindly provided by D. Chudakov, Shemyakin and Ovchinnikov Institute of Bioorganic Chemistry, Moscow, Russia) was amplified using primers 5′-ACTACCGGTCGTGTCTAAGGGCGAAGAG-3′ and 5′-ATCGCGGCCGCTTAATTAAGTTTGTGCCCCAG-3′. The PCR fragments were inserted into the AgeI/NotI sites of pLPS3'EGFP (Clontech, Mountain View, CA).

All SH2 domain containing constructs were cloned as PCR fragments into pDNR-Dual with the InFusion cloning-Kit and subsequently transferred to pEGFP-loxp or pGEX4T1-loxp by Cre/lox recombination. Clones encoding human cDNAs of Nck1 (hNck1, IMAGp958E061170Q2), Nck2 (hNck2, IMAGp958A12182Q), hCrkL (IMAGp998N1813292Q3), hGrb2 (IMAGp958J21133Q), hSLP-76 (IMAGp998E1110303Q3) and c-Src (IMAGp958B161238Q2) were obtained from Imagenes (Berlin, Germany). The SH2 domain of Nck1 (hNck1-SH2) was amplified using 5′-GAAGTTATCAGTCGACAAGTTTGCTGGCAATCCTTGG-3′ and 5′-ATGGTCTAGAAAGCTTCAGCAGTATCATGATAAATGCTTGAC-3′. Point mutation for hNck1R308K-SH2 was introduced by site-directed mutagenesis using primers 5′-GAAGGGGATTTCCTTATAAAAGATAGTGAATCTTCGCCAAATG-3′ and 5′-CGAAGATTCACTATCTTTTATAAGGAAATCCCCTTCATGTCC-3′. Full length hNck1 was amplified using 5′-GAAGTTATCAGTCGACATGGCAGAAGAAGTGGTGGTAG-3′ and 5′-ATGGTCTAGAAAGCTTCAGCAGTATCATGATAAATGCTTGAC-3′ and inserted into pDNR-Dual for subsequent recombination into pLP-CMV-myc (myc-Nck1) and pEGFP-loxp (GFP-Nck1). hNck2-SH2 domain was amplified using primers 5′-GAAGTTATCAGTCGACCCCTCGTCCAGCGG-3′ and 5′-ATGGTCTAGAAAGCTTACTGCAGGGCCCTGACG-3′, hGrb2-SH2 domain using primers 5′-GAAGTTATCAGTCGACCCCAAGAACTACATAGAAATG-3′ and 5′-ATGGTCTAGAAAGCTTAGTATGTCGGCTGCTGTG-3′, and hCrKL-SH2 domain using primers 5′-GAAGTTATCAGTCGACATGTCCTCCGCCAGGTTCG-3′ and 5′-ATGGTCTAGAAAGCTTGCAATCACTCCTTTTCATCTGGG-3′. Cloning of human c-Src and SLP-76 SH2 domains was described [18], [22].

Human WAVE2 cDNA was kindly provided by T. Stradal (Helmholtz Centre for Infection Research, Braunschweig, Germany). WAVE2 was amplified using primers WAVE2-IF-sense 5′- GAAGTTATCAGTCGACATGCCGTTAGTAACGAGGAACATCG-3′ and WAVE2-IF-anti 5′-ATGGTCTAGAAAGCTTTATTGGTCGGACCAGTCGTCCTC-3′. WAVE2ΔVCA was amplified using primers WAVE-IF-sense together with WAVE2ΔVCA-IF-anti 5′-ATGGTCTAGAAAGCTTTAGGCATCGCTCACGGCAGGC-3′. PCR fragments were cloned into pDNR-dual and the transferred by Cre/lox recombination into pEGFP-loxp (GFP-WAVE2-WT, GFP-WAVE2-ΔVCA) or pLP-CMV-myc (myc-WAVE2-WT, myc-WAVE2-ΔVCA), respectively.

### Antibodies and reagents

Monoclonal antibody (mAb) against CEACAMs (clone IH4Fc) was from Immunotools (Friesoythe, Germany), mAb against GFP (clone JL-8) was from Clontech (Mountain View, CA), mAbs against v-Src (clone EC10) and against phospho-tyrosine (4G10) were from Upstate Biotechnology (Lake Placid, NY), mAb against GST (clone B-14) was from Santa Cruz Biotechnology (Santa Cruz, CA). Monoclonal antibody against Nck1/2 (clone 108) was from BD Transduction Laboratories (Heidelberg, Germany), rabbit monoclonal antibody against WAVE (clone D2C8) was from Cell Signalling Technology (Danvers, MA). Polyclonal rabbit antibody against GFP was produced in the local animal facility at the University of Konstanz, rabbit polyclonal antibody against Nap1 (Hem2, NckAP1) was from Abcam (Cambridge, UK). Polyclonal antibody IG511 against pathogenic Neisseria has been described previously [22]. MAbs against the HA-tag (clone 12CA5), myc-tag (clone 9E10), and tubulin (clone E-7) were purified from hybridoma cell supernatants obtained from DSHB (University of Iowa, IA). GST and GST-fused SH2-domains used were all expressed in *E. coli* BL-21 and purified using GSTrap FF (Amersham Biosciences, Freiburg, Germany). Protein A/G sepharose was obtained from Santa Cruz Biotechnology. Streptavidin-Alexafluor647 and Phalloidin-Alexafluor546 were obtained from Molecular Probes/Invitrogen (Carlsbad, CA). Secondary antibodies were obtained from Jackson Immunoresearch (West Grove, PA). NHS-Biotin was obtained from Pierce Biotechnology (Rockford, IL).

### Transfection of cells, cell lysis and Western blotting

293 cells were transfected by calcium phosphate precipitation using 3 µg of CEACAM constructs or empty vector control. For co-transfection, 5 µg of co-transfected constructs together with 3 µg of CEACAM constructs were used and in all samples total DNA was adjusted to 8 µg using the empty control vector. Cell lysis and Western blotting were performed as described [15]. CEACAM3-expressing HeLa cells were transfected with a combination of two distinct siRNAs directed against human Nck1 at 5 nM (Mission predesigned siRNA, Sigma Aldrich, St. Louis, MO) using Turbofect reagent (Fermentas, St. Leon-Rot, Germany) according to manufacturers protocol. For plasmid transfection, HeLa cells and murine fibroblasts were transfected with Lipofectamin 2000 plus according to manufacturers recommendations (Invitrogen, Carlsbad, CA) and used in assays 48 h after transfection.

### Gentamicin protection assay

Gentamicin protection assays were conducted as described [15]. Cells were seeded in gelatine-coated 48 well dishes at 2.5×10^5^ cells/well. A multiplicity of infection of 20 bacteria per cell was routinely used and after 30 minutes of infection, extracellular bacteria were killed by 45 min incubation in 50 µg/ml gentamicin in DMEM. Binding of bacteria to the cells was determined after 30 min of infection and three washes with PBS. Cells were lysed with 1% saponin in PBS for 15 min. The samples were diluted with PBS and the number of viable bacteria was determined by plating suitable dilutions in duplicate on vitamin supplemented GC-agar.

### GST-pull down and immunoprecipitation

For GST-pull downs, 3 µg of purified GST or GST-fusion protein attached to glutathione-sepharose were added to WCLs and incubated for 4 h at 4°C. After four washes with RIPA buffer, precipitates were boiled in SDS sample buffer, before SDS-PAGE and Western blot analysis. For co-immunoprecipitations, 293 cells were transfected with the indicated combination of constructs and lysed after 48 h. For precipitation, lysates were incubated with 3 µg of polyclonal rabbit anti-GFP antibody over night followed by 1 h incubation with protein A/G sepharose, all at 4°C. After three washes with Triton buffer, precipitates were boiled in SDS sample buffer before SDS-PAGE and Western blot analysis.

### Immunofluorescence staining

Cells transfected with the indicated constructs were grown on glass coverslips in 24-well plates and infected for 30 min with PacificBlue-labelled and biotinylated (NHS-Biotin) Opa_CEA_-expressing *N. gonorrhoeae* at an MOI of 40. Samples were fixed with 4% paraformaldehyde in PBS and washed three times with PBS++ (PBS with additional Ca^2+^ and Mg^2+^), prior to incubation in blocking buffer (PBS++, 10% FCS) for five minutes. Biotinylated bacteria were stained with streptavidin-Alexafluor647 prior to permeabilization. Epitope-tagged constructs were visualized in a 2-step process including detection with specific mAbs (against HA-tag or myc-tag) and staining with Cy5-coupled secondary antibody (goat-anti-mouse-Cy5). Samples were rinsed twice with PBS++ and cells permeabilized with blocking buffer containing 0.2% saponin before incubation with antibodies. Visualisation of the actin cytoskeleton was achieved using Phalloidin-Alexafluor546. Samples were viewed with a Leica SP5 laser scanning confocal microscope (Leica, Wetzlar, Germany) using a 63×, 1.3 NA Plan Neofluar oil-immersion objective. Fluorescence signals of multicolored specimens were serially recorded with appropriate excitation and emission settings to avoid bleed-through. Images were digitally processed with ImageJ and merged to yield pseudo-coloured pictures.

### Correlative light and electron microscopy (CLEM)

Nck1+/−Nck2+/−cells or Nck1−/−Nck2−/−cells were transfected with CEACAM3-GFP encoding plasmid. Transfected cells were grown on CeLLocate coverslips (Eppendorf, Hamburg, Germany) in 24-well plates and infected for 30 min with PacificBlue-labelled Opa_CEA_-expressing *N. gonorrhoeae* at an MOI of 40. Samples were fixed overnight at 4°C in 2% glutaraldehyde, 3% formaldehyde in 0.1 M sodium-cacodylate buffer (pH 6.9) containing 0.09 M sucrose, 0.01 M CaCl_2_, 0.01 M MgCl_2_. The fixed samples were washed with 0.1 M sodium-cacodylate buffer and quickly analyzed with a Leica SP5 laser scanning confocal microscope (Leica, Wetzlar, Germany) as described above. Subsequently, the samples were dehydrated in a graded series of ethanol followed by critical point drying with CO_2_ and sputter-coating with 5 nm gold-palladium in a BAL-TEC SCD 030. The regions of interest were re-located according to the grid on CeLLocate coverslips and analyzed on a Zeiss Auriga CrossBeam Workstation operating at 15 kV using the secondary electron detector. Correlation of confocal and SEM images was achieved using Adobe Photoshop CS5.

### shRNA construction and lentiviral production

Recombinant lentiviral particles were generated using the plasmids pLKO.1, pLKO.1-shScrambled (containing a non-targeting control shRNA-sequence), pMD2.G, and psPAX2 provided by Addgene (www.addgene.org) and maintained in *E. coli* STBL4 (Invitrogen, Carlsbad, CA). Using the algorithm AAGN_18_TT (available online at http://jura.wi.mit.edu/bioc/siRNAext/) we identified sequences that could silence expression of human WAVE2 or Nap1 (NCKAP1, HEM2), respectively. According to this prediction, complementary primers were synthesized: hWAVE2-shRNA-sense 5′- CCGGAAGCAACCAAAGATATATCCACTCGAGTGGATATATCTTTGGTTGCTTTTTTTG-3′ and hWAVE2-shRNA-anti 5′- AATTCAAAAAAAGCAACCAAAGATATATCCACTCGAGTGGATATATCTTTGGTTGCTT-3′; Nap1: hNap1-shRNA-sense 5′-CCGGAAGAGCAGCAGCATATGTTGTCTCGAGACAACATATGCTGCTGCTCTTTTTTTG-3′ and hNap1-shRNA-anti 5′-AATTCAAAAAAAGAGCAGCAGCATATGTTGTCTCGAGACAACATATGCTGCTGCTCTT-3′. The oligos were annealed and cloned into the AgeI and EcoRI site of pLKO.1 generating pLKO.1-shWAVE2 and pLKO.1-shNap1, respectively. The correct insertion of the shRNA cassette was verified by sequencing.

For lentiviral production, 2×10^6^ 293 cells were transiently transfected with 14 µg of the respective pLKO.1 vector together with 10 µg of packaging plasmid psPAX2 and 7 µg of envelope-coding plasmid pMD2.G. 48 h later, the virus-containing cell culture supernatant was collected, centrifuged at 2000 rpm at 4°C for 7 min and filtered through a 0.45 µm pore-size filter. 5 ml of the cleared viral supernatant was used to transduce 293 cells in 10 cm culture dishes. After 24 h, puromycin (0.4 µg/ml) was added and the puromycin-resistant stable cell population was used in experiments after 6 days of selection.

### Quantification of bacterial uptake by flow cytometry

Uptake of bacteria was quantified by flow cytometric analysis as described previously [25]. Briefly, 293 cells with stable knock down of WAVE2, HEM2 or control transduced cells (shScramble) were transfected with HA-tagged CEACAM3 and 1×10^6^ of the transfected cells were seeded in 6 well-plates the day before infection. Cells were infected at a MOI (multiplicity of infection) of 20 with CFSE-labeled non-opaque or Opa_CEA_-expressing *N. gonorrhoeae* for 1 h. After infection, cells were suspended in PBS, 1% heat-inactivated FCS, 2 mg/ml trypan blue and analysed on a LSRII (Becton Dickinson). The percentage of fluorescein-positive cells was multiplied by the mean fluorescence of these cells to obtain an estimate of the total amount of phagocytosed bacteria (uptake index). In parallel, CEACAM expression by the transfected knock-down cells was determined by flow cytometry with a monoclonal anti-CEACAM antibody. The mean fluorescence intensity of CEACAM3 staining was used to normalize the uptake index to correct for differences in the transfection efficiency of the three knock-down cell populations.

## Results

### The cytoplasmic domain of CEACAM3 associates with the adaptor protein Nck

**Figure 1:**
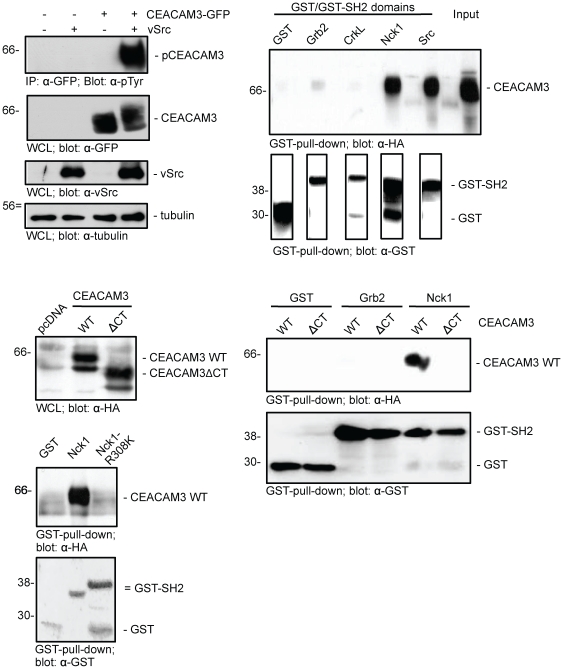
The SH2 domain of Nck1 associates with the cytoplasmatic domain of CEACAM3. (A) 293 cells were transfected with a plasmid encoding GFP- and HA-tagged CEACAM3 (CEACAM3-GFP) or GFP alone and co-transfected or not with v-Src. CEACAM3 was immunoprecipitated (IP) from lysates and the phosphorylation status was assayed by Western blotting with monoclonal antibodies against phospho-tyrosine (pTyr)(upper panel). The expression of the proteins was verified by probing whole cell lysates (WCL) with antibodies against GFP, vSrc, or tubulin (lower panels). (B) The indicated GST-SH2 domains or GST alone were used in pulldown assays with lysates from cells co-expressing CEACAM3-GFP and vSrc as in (A). Precipitates were analysed by Western blot with monoclonal HA-tag antibody to detect SH2 domain-bound CEACAM3 (upper panel). Input shows CEACAM3 expression in 1/10 of the lysate used for pulldown. Membranes were stripped and re-probed with GST antibodies to detect the GST fusion proteins (lower panel). (C) 293 cells were transfected with GFP- and HA-tagged wildtype CEACAM3 (CEACAM3 WT), a CEACAM3 mutant with a deletion of the complete cytoplasmatic domain (CEACAM3 ΔCT), or an empty vector (pcDNA) and co-transfected with v-Src. The expression of the receptor proteins was verified by probing whole cell lysates (WCL) with an antibody against the HA-tag. (D) Lysates from (C) were used in pulldown assays with GST, GST-Grb2-SH2, or GST-Nck1-SH2. Precipitates were analysed by Western blotting as in (B). (E) Lysates from cells co-expressing CEACAM3-GFP and vSrc were precipitated with GST, GST-Nck1-SH2, or a mutant of the Nck1 SH2 domain that is unable to bind phospho-tyrosine (GST-Nck1-R308K). Precipitates were analysed as in (B).

Phosphotyrosine-based signalling complexes often involve SH2-domain containing adapter molecules. To identify adapter molecules potentially interacting with the ITAM-like sequence of CEACAM3, we examined a panel of SH2 domains derived from different adaptor proteins for their capacity to interact with phosphorylated CEACAM3. In a pull-down assay, GST-fusion proteins of SH2 domains derived from Grb2, CrkL, Nck1, or, as a positive control, the PTK c-Src were added to cell lysates containing phosphorylated full-length CEACAM3. Phosphorylation of CEACAM3 in the lysates was ensured by co-expression of vSrc, a constitutive active viral form of the cellular PTK c-Src, which phosphorylates CEACAM3 at tyrosine residues in the cytoplasmic ITAM-like sequence [18], [20] (Fig. 1A). As expected, the recombinant c-Src SH2 domain fused to GST precipitated CEACAM3 from the lysate (Fig. 1B). This is in line with previous reports that show co-localization and a direct association of Src family PTK SH2 domains with tyrosine phosphorylated CEACAM3 [18], [21]. In contrast, GST alone or the SH2 domains of the adaptor proteins CrkL and Grb2 were not able to precipitate CEACAM3. Importantly, the SH2 domain of Nck1 interacted strongly with CEACAM3 (Fig. 1B). To analyse if the specific binding of Nck1 involves the cytoplasmic domain of CEACAM3, additional pull-down assays were performed using full-length CEACAM3 (CEACAM3 WT) and a deletion mutant of the receptor that lacks the complete cytoplasmic domain (CEACAM3 ΔCT). Upon transfection of 293 cells and preparation of lysates, CEACAM3 WT or CEACAM3 ΔCT were present at similar levels (Fig. 1C). However, the Nck1 SH2 domain associated only with full-length, but not with truncated CEACAM3 (Fig. 1D). Again, the association was specific for the SH2 domain of Nck1 and was not observed for GST alone or the SH2 domain of Grb2 (Fig. 1D).

SH2-domain-mediated interactions require an intact phospho-tyrosine binding pocket. In Nck1, a crucial arginine residue is located in this binding pocket at position 308 (R308) of the full length Nck1 protein. Accordingly, we performed site-directed mutagenesis and replaced R308 for lysine. In contrast to the wildtype Nck1 SH2 domain, the resulting Nck1-R308K-SH2 domain lost its ability to associate with CEACAM3 (Fig. 1E), demonstrating that the observed interaction is mediated by a phospho-tyrosine-directed binding of the Nck1 SH2 domain.

### Nck1 and Nck2 bind to the phosphorylated cytoplasmatic domain of CEACAM3

**Figure 2:**
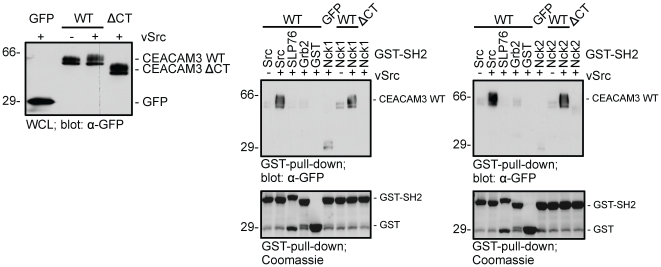
Nck1 and Nck2 bind to the tyrosine phosphorylated cytoplasmatic domain of CEACAM3. (A) 293 cells were transfected with plasmids encoding GFP- and HA-tagged CEACAM3 wild type (WT), CEACAM3 ΔCT, or GFP and cotransfected or not with vSrc. Whole cell lysates (WCL) were analysed by Western blot with monoclonal anti-GFP antibody. (B) Lysates as in (A) were precipitated with the indicated GST-SH2-domain fusion proteins or GST alone. Precipitates were analysed by Western blotting with monoclonal GFP antibody to detect precipitated CEACAM3 (upper panels). The membranes were stained with Coomassie Brillliant Blue (Coomassie) to verify equal amounts of GST or GST-fusion proteins in the precipitates (lower panel).

In humans, two Nck homologues, Nck1 and Nck2, are expressed in most cell types [25]. To analyze the ability of either of these two proteins to associate with CEACAM3, we employed GST fusion proteins of the SH2 domains of either Nck1 or Nck2. Lysates were prepared from 293 cells transfected with GFP, CEACAM3 WT-GFP, or CEACAM3 ΔCT-GFP in the presence or absence of v-Src (Fig. 2A). Using these lysates in pull-down analyses, both the Nck1 as well as the Nck2 SH2 domain were able to bind to full-length CEACAM3, but not to CEACAM3 ΔCT (Fig. 2B). Again, binding of the Nck1 and Nck2 SH2 domains to CEACAM3 WT was comparable to the interaction found for the c-Src SH2 domain (Fig. 2B). In contrast to Nck, the SH2 domains of the two adapter proteins SLP76 and Grb2, or GST alone did not associate with CEACAM3 (Fig. 2B). Importantly, association of Nck1 and Nck2 with CEACAM3 depended on tyrosine phosphorylation of the cytoplasmic domain, as binding did not occur, if CEACAM3 was not co-expressed with vSrc (Fig. 2A). In all cases, GST and GST-SH2 domains were employed in comparable amounts as shown by Coomassie staining of the blotted precipitates (Fig. 2B, lower panels). Together, these results demonstrate that both Nck1 and Nck2 SH domains can specifically bind to the phosphorylated cytoplasmatic domain of CEACAM3.

### CEACAM3 and Nck1 co-localize at sites of bacterial attachment

**Figure 3:**
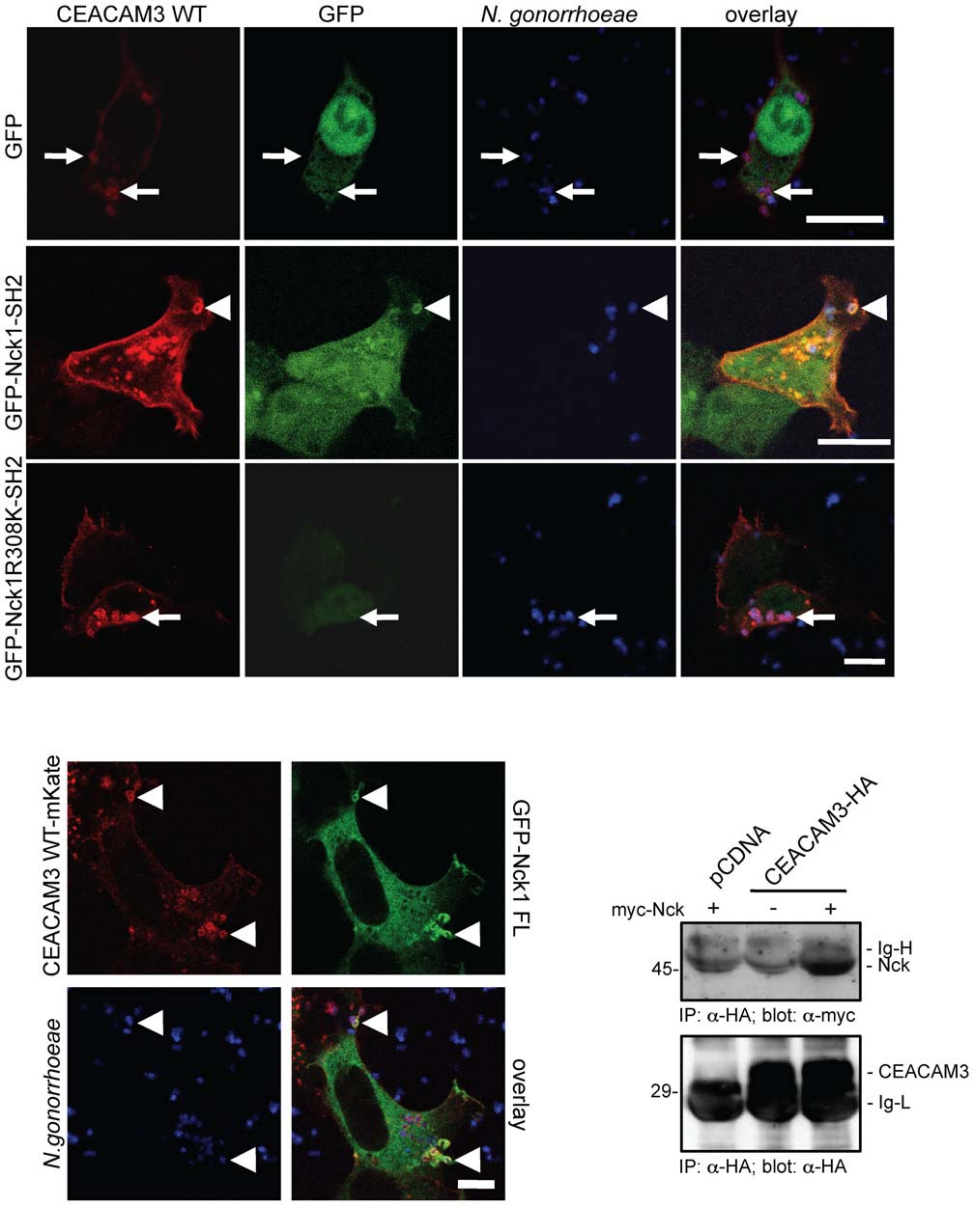
Association of Nck1 and CEACAM3 occurs in intact cells upon *N. gonorrhoeae* infection. (A) 293 cells were transfected with plasmids encoding mKate-tagged CEACAM3 WT (red) and co-transfected with either GFP, GFP-Nck1-SH2, or GFP-Nck1-R308K-SH2 (green). Cells were infected for 30 minutes with PacificBlue-labelled Opa_CEA_-expressing *N. gonorrhoeae* (blue). Fixed samples were analysed by confocal laser scanning microscopy. Bacteria cluster CEACAM3 and induce recruitment of Nck1-SH2 (arrowhead), but not GFP or Nck1-R308K-SH2 (small arrows). Bars indicate 20 µm. (B) 293 cells were transfected with mKate-tagged CEACAM3 WT (red) and co-transfected with full-length GFP-Nck1 (GFP-Nck1; green). Samples were infected for 30 minutes with Opa_CEA_-expressing *N. gonorrhoeae* (blue) and, after fixation, bacteria were stained with polyclonal antibody and Cy5-coupled secondary reagents. Confocal laser scanning microscopy revealed that Nck was strongly enriched at sites of bacterial contact with CEACAM3 (arrowheads). Bars indicate 5 µm. (C) 293 cells were transfected with plasmids encoding vSrc together with pcDNA or HA-tagged CEACAM3 WT. Where indicated, cell were co-transfected with full-length myc-tagged Nck1. After lysis, CEACAM3 WT was immunoprecipitated (IP) with mAb against the HA-epitope. After Western blotting, precipitates were probed with mAb against myc-Nck1 (upper panel) and, after stripping of the membrane against the immunoprecipitated CEACAM3 WT with mAb against the HA-tag (lower panel). The immunoglobulin heavy (Ig-H) and light chain (Ig-L) of the precipitating antibody are indicated.

Upon infection with *Neisseria gonorrhoeae*, CEACAM3 clustering triggers recruitment of Src-family PTKs to the bacteria-engaged receptor [21]. To localize the potential interaction of Nck with the bacteria-associated receptor, we co-transfected 293 cells with constructs encoding the red-fluorescent protein mKate fused to the carboxy-terminus of wildtype CEACAM3 (CEACAM3-mKate) together with either GFP, the GFP-tagged Nck1 SH2 domain (GFP-Nck1-SH2) or the GFP-tagged, mutated Nck1 SH2 domain (GFP-Nck1R308K-SH2). All constructs were successfully expressed at moderate levels suitable for analysis by confocal laser scanning microscopy. As expected, CEACAM3-mKate was predominantly found in cellular membranes (Fig. 3A). Following infection with CEACAM-binding gonococci, CEACAM3-mKate clustered at sites of bacterial engagement, but this did not result in re-distribution of GFP (Fig. 3A). Clearly, the GFP-fused Nck1 SH2 domain was strongly recruited to the contact sites of CEACAM3 and bacteria (Fig. 3A; arrowhead). This recruitment was evident for bacteria during the initial uptake process in the cell periphery, but not for bacteria in a perinuclear, intracellular position suggesting that Nck recruitment is transient. In contrast to the wildtype Nck1 SH2 domain, Nck1R308K-SH2, which did not interact with phosphorylated CEACAM3, did not accumulate in response to bacterial CEACAM3 engagement and remained evenly distributed throughout the cell (Fig. 3A). In line with the selective recruitment of the isolated Nck1 SH2 domain, also GFP-tagged full length Nck1 (GFP Nck1 FL) was strongly recruited to sites of bacterial attachment in 293 cells expressing a red fluorescent protein tagged CEACAM3 (Fig. 3B). The formation of a complex between Nck1 and CEACAM3 in intact cells was verified by co-immunoprecipitation of full length Nck1 together with CEACAM3 from cell lysates with co-expression of vSrc (Fig. 3C). These results provide evidence that a CEACAM3-Nck complex is formed in intact cells upon bacterial engagement of CEACAM3 and suggest that Nck could be involved in orchestrating the opsonin-independent phagocytosis of CEACAM3-bound microbes.

### Overexpression of the Nck SH2 domain or knock-down of Nck1 reduce bacterial uptake via CEACAM3

**Figure 4:**
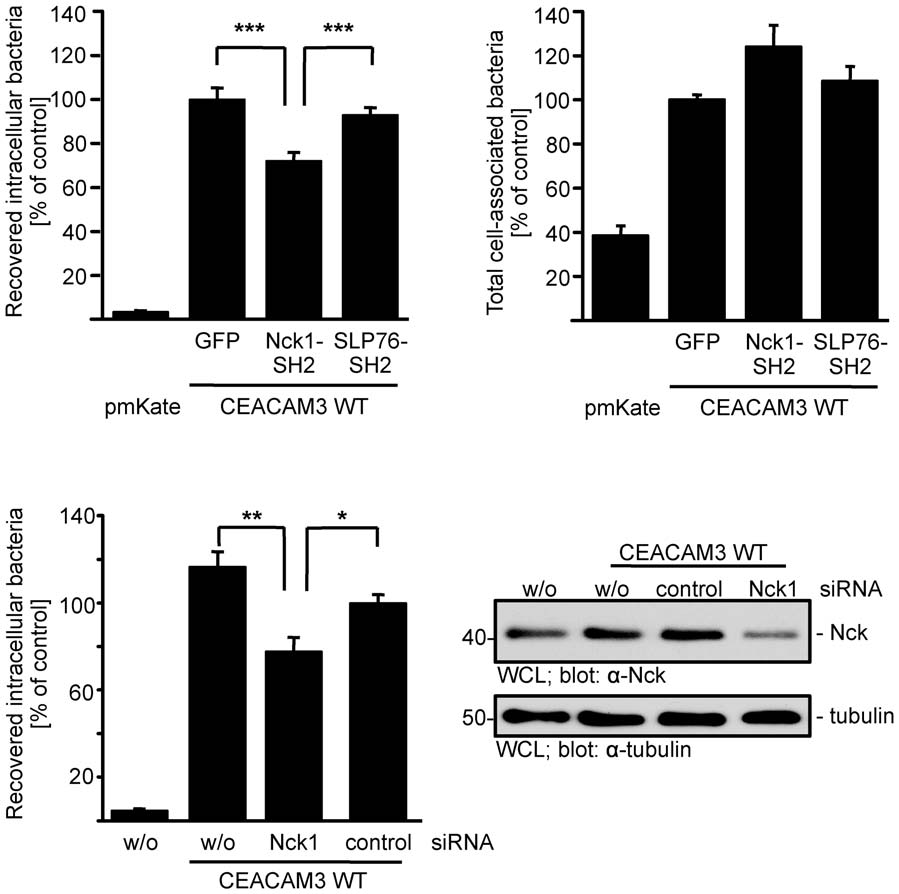
Nck has a functional role in CEACAM3-mediated uptake of bacteria. (A) 293 cells were transfected with constructs encoding mKate or mKate-tagged CEACAM3 WT and co-transfected with either GFP alone, GFP-Nck1-SH2 or YFP-SLP76-SH2. Transfected cells were infected for 30 minutes with Opa_CEA_-expressing *N. gonorrhoeae* and employed in a gentamicin protection assay. Bars represent mean values ± S.E.M of four independent experiments done in triplicate. Significance was tested using a paired, two-sided Student's t-test; ***, p<0.001. (B) Cells were transfected and infected as in (A) and employed in a bacterial adhesion assay. Bars represent mean values ± S.E.M of four independent experiments done in triplicate. (C) HeLa cells stably expressing CEACAM3 WT were transfected with Nck1-siRNA, an unspecific control siRNA, or left untransfected (w/o). 72 h after transfection, cells were infected for 30 minutes with Opa_CEA_-expressing *N. gonorrhoeae* and employed in a gentamicin protection assay. Bars represent mean values ± S.E.M of four independent experiments done in triplicate. Significance was tested using a paired, two-sided Student's t-test; **, p<0.01, *, p<0.05. Whole cell lysates (WCL) of the transfected cells were analysed by Western blotting with mAB against Nck-1 (upper panel) or against tubulin (lower panel).

If the observed specific recruitment of Nck1 to clustered CEACAM3 would have a functional role for bacterial internalization, then blocking access of Nck to the cytoplasmic domain of the receptor should interfere with the uptake process. Therefore, we overexpressed the isolated Nck1 SH2 domain or the SLP-76 SH2 domain together with CEACAM3 and monitored bacterial internalization by gentamicin protection assays. In line with the idea that association of Nck1 with CEACAM3 is needed for efficient internalization of bacteria, overexpression of the Nck1, but not the SLP-76 SH2 domain reduced the uptake of CEACAM-binding gonococci significantly by about 25% (Fig. 4A). In contrast, adhesion of bacteria to CEACAM3-expressing cells was not diminished in the presence of the Nck1 SH2 domain (Fig. 4B). Though these results suggest that Nck is involved in the CEACAM3-mediated uptake of *N. gonorrhoeae*, the isolated SH2 domain of Nck1 could interfere with other SH2 domain-dependent processes such as recruitment of Src family PTKs to the cytoplasmic domain of the receptor. Therefore, we depleted endogenous Nck1 by siRNA in CEACAM3-expressing HeLa cells. Knock-down of Nck1 was only partial and reduced the amount of Nck1 by about 60% (Fig. 4C). However, this partial reduction led to a significant decrease in CEACAM3-mediated uptake of gonococci compared to untreated or control siRNA-treated cells (Fig. 4C). As a further control, HeLa cells, that do not express any CEACAM member endogenously, did not internalize gonococci (Fig. 4C). Together with the biochemical and microscopic analysis, these results identify a functional role for the adapter molecule Nck in regulating the CEACAM3-initiated uptake of bacteria.

### Genetic deletion of Nck1 and Nck2 interferes with CEACAM3-mediated uptake

**Figure 5:**
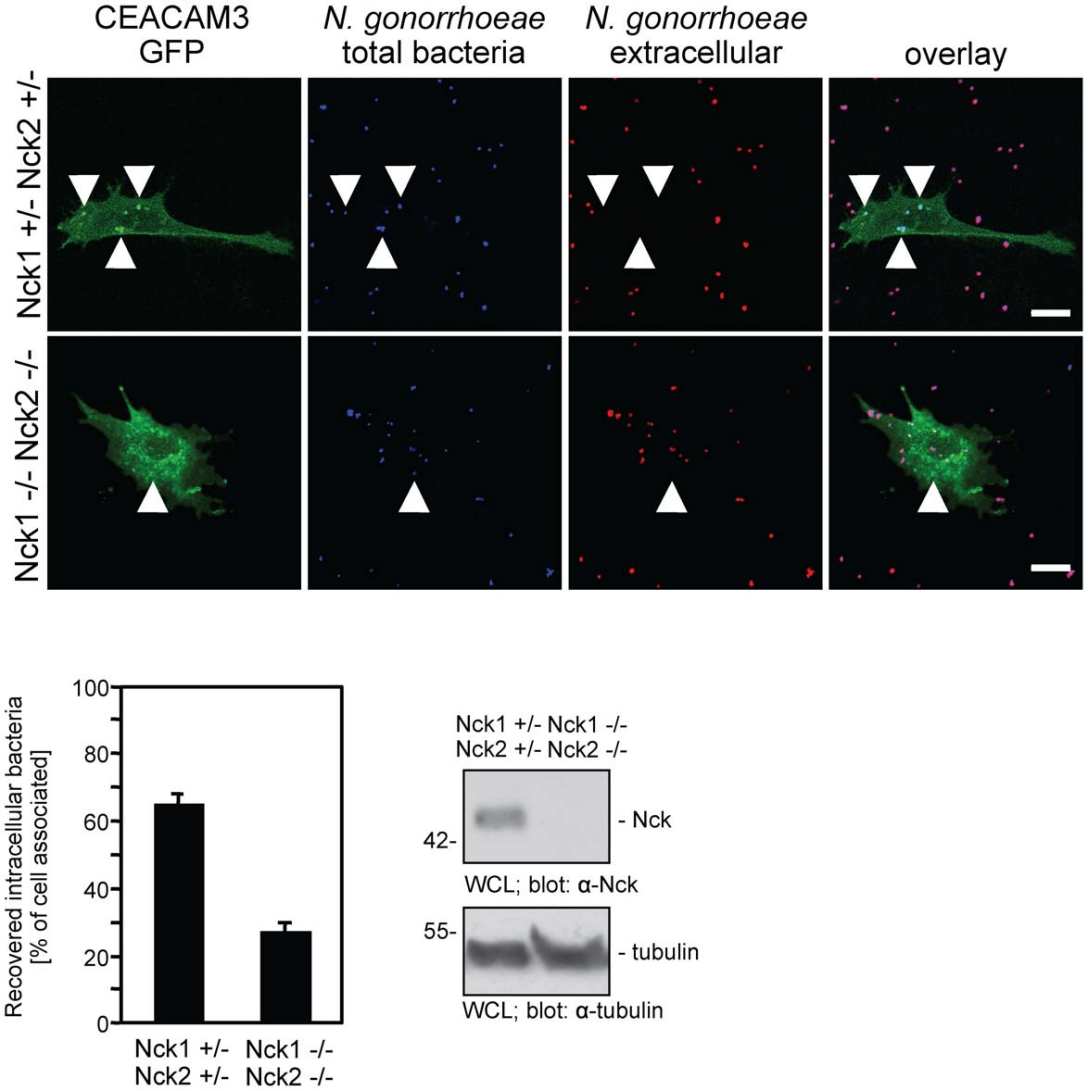
Nck1-Nck2-deficient fibroblasts show diminished CEACAM3-mediated uptake of bacteria. (A) Nck1/Nck2-deficient mouse fibroblasts (Nck1−/− Nck2−/−) and Nck1 and Nck2-expressing fibroblasts (Nck1+/− Nck2+/−) were transfected with a plasmid encoding GFP-tagged CEACAM3 and infected with biotinylated, Pacific blue-labelled Opa_CEA_-expressing *N. gonorrhoeae*. 60 minutes after infection, extracellular bacteria were stained with streptavidin-Alexafluor647 allowing detection of intracellular bacteria by their selective Pacific blue staining (arrowheads). (B) Total cell-associated and intracellular bacteria were quantified in cells stained as in (A). Bars represent the mean values ± S.E.M of intracellular bacteria (n = 30 cells). (C) Whole cell lysates (WCL) of cells used in (A) were probed with a monoclonal anti-Nck1/Nck2 antibody (top panel) or a monoclonal anti-tubulin antibody (lower panel).

In most cells, Nck1 and Nck2 have overlapping functions. Therefore, we employed fibroblasts derived from homozygote Nck1/Nck2-deficient mouse embryos (Nck1−/−Nck2−/−cells) and compared them to heterozygote control cells (derived from Nck1+/−Nck2+/−mouse embryos) [24]. The fibroblasts were transfected with a plasmid encoding GFP-tagged CEACAM3 and infected with biotinylated, Pacific blue-labelled Opa_CEA_-expressing *N. gonorrhoeae*. After 60 minutes infection, the fixed samples were stained for extracellular bacteria with streptavidin- Alexafluor647 allowing detection of cell-associated versus intracellular bacteria (Fig. 5A). Though intracellular bacteria were found in all CEACAM3-expressing fibroblasts, the amount of intracellular bacteria in Nck1−/−Nck2−/−cells was strongly diminished compared to Nck1+/−Nck2+/−cells (Fig. 5A). Quantification of intracellular bacteria revealed that Nck-deficiency reduced bacterial uptake by about 60% (Fig. 5B). Western blotting confirmed the lack of intact Nck1 and Nck2 in cell lysates of Nck1−/−Nck2−/−cells (Fig. 5C). Together, these data support the idea that the adaptor proteins Nck1 and Nck2 contribute to the efficient CEACAM3-mediated phagocytosis of bacteria.

### Nck1 and Nck2-deficient cells lack CEACAM3-initiated lamellipodia

**Figure 6:**
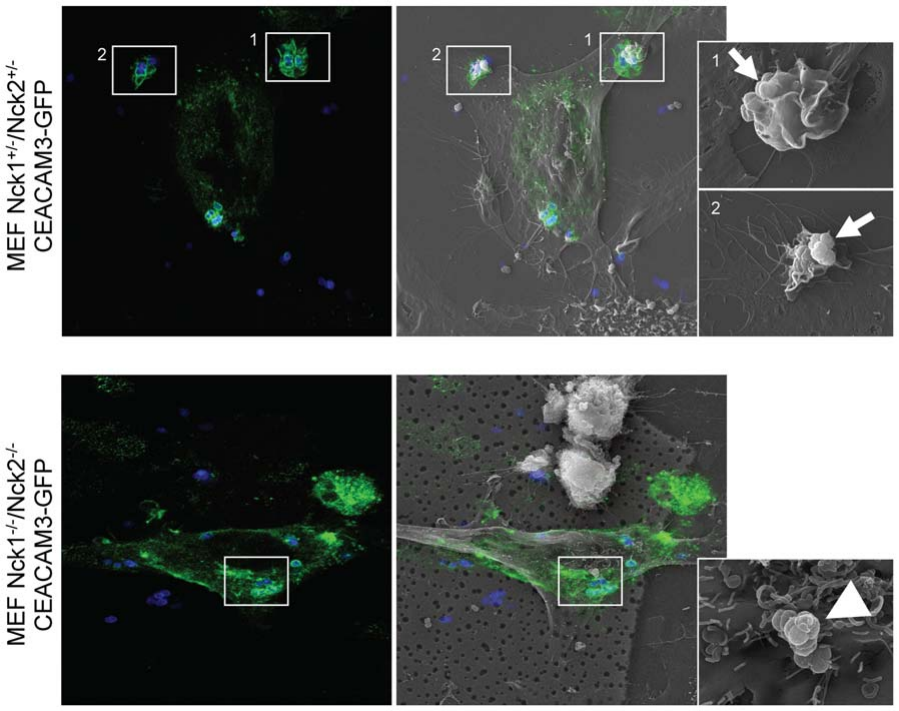
Nck1 and Nck2-deficient cells lack CEACAM3-initiated lamellipodia. Nck1 and Nck2-expressing fibroblasts (Nck1+/−/Nck2+/−, upper panel) and Nck1/Nck2-deficient mouse fibroblasts (Nck1−/−/Nck2−/−, lower panel) were transfected with a plasmid encoding GFP-tagged CEACAM3 and infected with Pacific blue-labelled Opa_CEA_-expressing *N. gonorrhoeae* for 30 minutes. Fixed samples were first analyzed by confocal laser-scanning microscopy and the identical cells were imaged in a second step by scanning electron microscopy (SEM). Confocal images (left) showing CEACAM3-transfected cells (green) with bound gonococci (blue) were overlayed with the SEM image of the same cell (middle) allowing identification of subcellular areas of CEACAM3-mediated bacterial engulfment (white boxes). Higher resolution SEM images of the boxed areas demonstrate extensive lamellipodial protrusions in Nck-expressing cells (small arrows), whereas CEACAM3-engagement in Nck-deficient cells does not elicit lamellipodia in the vicinity of bound bacteria (arrowhead).

Nck adapter proteins are often help to locally organize dynamic changes in the actin cytoskeleton upon stimulation of membrane receptors. As CEACAM3-initiated uptake of bacteria is accompanied by actin re-arrangements leading to large lamellipodial membrane protrusions [15], [16], we wondered whether Nck adapter proteins might be critical for this process. To address this question, we implemented correlative light-electron microscopy (CLEM) to visualize lamellipodial structures in response to bacterial CEACAM3 stimulation in the presence or absence of Nck adapter proteins. To this end, Nck1+/−Nck2+/−and Nck1−/−Nck2−/−cells were transiently transfected with CEACAM-GFP and infected with fluorescence labelled Opa_CEA_ protein-expressing gonococci. Both, Nck-expressing and Nck-deficient fibroblasts bound equal numbers of gonococci and the Opa_CEA_ protein-expressing bacteria led to clustering of CEACAM3-GFP, as visualized by fluorescence microscopy (Fig. 6). Upon further processing, the identical cells were re-analysed by scanning electron microscopy allowing high resolution observation of surface structures (Fig. 6). Whereas CEACAM3-bound bacteria on the surface of Nck1−/−Nck2−/−cells were surrounded by small filopodia, large lamellipodial protrusions were evident in a radius up to 6 µm in the vicinity of gonococci in contact with CEACAM3 on Nck-expressing fibroblasts (Fig. 6). Therefore, this CLEM investigation provides direct evidence that Nck adapter proteins are involved in CEACAM3-initiated, actin cytoskeleton-based subcellular structures.

### CEACAM3 associates with a Nck- and WAVE-containing multiprotein complex upon bacterial engagement

**Figure 7:**
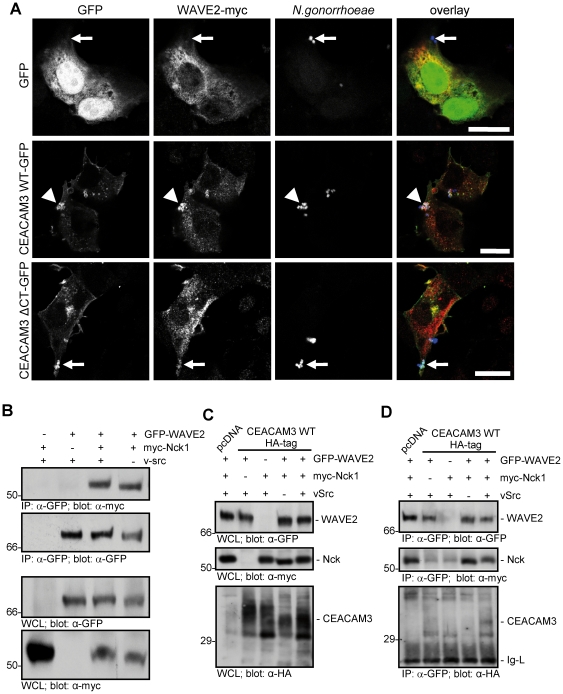
Nck recruits the WAVE2 complex to the phosphorylated cytoplasmatic domain of CEACAM3. (A) 293 cells were transfected with CEACAM3 WT-GFP, CEACAM3 ΔCT-GFP or GFP alone (green) and co-transfected with myc-WAVE2 (red). Samples were infected for 30 minutes with PacificBlue-labelled Opa_CEA_-expressing *N. gonorrhoeae* (blue), and, after fixation, myc-WAVE2 was detected using mAb myc and Cy5-goat-anti-mouse secondary antibody. Confocal laser scanning microscopy revealed the recruitment of WAVE2 to sites of bacteria-induced CEACAM3 WT clustering (arrowheads), whereas CEACAM3 ΔCT or GFP did not induce major relocation of WAVE2 (small arrows). Bars indicate 20 µm. (B) 293 cells were transfected with GFP-WAVE2, myc-Nck1, and vSrc as indicated. GFP-WAVE2 was immunoprecipitated (IP) with rabbit polyclonal GFP-antibody and precipitates (upper panels) as well as WCLs (lower panels) were analysed by Western blot with mAb against the myc-tag or against GFP. (C) 293 cells were co-transfected with GFP-WAVE2, myc-Nck1, CEACAM3 WT-HA and vSrc as indicated. WCLs were analysed by Western blotting with mAb against GFP, the myc-tag, or the HA-tag. (D) GFP-WAVE2 was immunoprecipitated (IP) from lysates generated in (C) and precipitates were analysed as in (C). The immunoglobulin light chain (Ig-L) of the precipitating antibody is indicated.

CEACAM3-mediated uptake of bacteria is strictly dependent on actin polymerisation orchestrated by the small GTPase Rac [15]. A well characterized effector of GTP-bound Rac is the WAVE/Scar-complex that promotes f-actin nucleation by the Arp2/3 complex [26], [27]. One critical component of the WAVE complex, Nap1 (Nck-associated protein 1, also termed NCKAP1 or Hem2), is a known binding partner of the SH3 domain of Nck. Giving the role of Nck in CEACAM3-initiated lamellipodia formation, we wondered whether Nck could help to localize the WAVE complex to CEACAM3. Accordingly, 293 cells were transfected with GFP, CEACAM3 WT-GFP, or CEACAM3 ΔCT-GFP together with myc-tagged WAVE2 and then infected with CEACAM-binding gonococci. Confocal microscopy revealed that WAVE2 was distributed throughout the cytoplasm in cells expressing GFP irrespective of bacterial infection (Fig. 7A). However, WAVE2 was strongly recruited to sites of bacterial engagement of CEACAM3 in cells expressing the wildtype receptor (Fig. 7A). In contrast, WAVE2 was not redistributed in cells expressing truncated CEACAM3 (CEACAM3 ΔCT-GFP), even though CEACAM3 ΔCT-GFP bound bacteria similar to the wildtype receptor (Fig. 7A). Immunoprecipitation of WAVE2 from cells cotransfected or not with myc-tagged Nck and vSrc revealed a constitutive association of WAVE2 and Nck, which was not dependent on the presence of vSrc (Fig. 7B). This is in line with the idea that Nck binds to the WAVE complex in a phosphotyrosine-independent, SH3 domain-dependent manner.

To test the possibility that Nck connects phosphorylated CEACAM3 with the WAVE complex, we transfected 293 cells with combinations of CEACAM3-WT-HA, GFP-WAVE2, myc-Nck1, and vSrc. Expression of all the constructs at equivalent levels was verified by Western blotting (Fig. 7C). Upon immunoprecipitation of GFP-WAVE2, the resulting precipitates were probed for the presence of myc-Nck and CEACAM3-HA (Fig. 7D). Again, a constitutive association of Nck with WAVE2 was detected, which did not depend on the presence of CEACAM3 or v-Src (Fig. 7D). However, CEACAM3 WT was only present in WAVE2 precipitates, if the cells co-expressed myc-Nck and vSrc demonstrating that Nck orchestrates the complex formation between tyrosine phosphorylated CEACAM3 and the Rac effector WAVE2 (Fig. 7D).

### Overexpression of dominant-negative WAVE2 impairs the phagocytic function of CEACAM3

**Figure 8:**
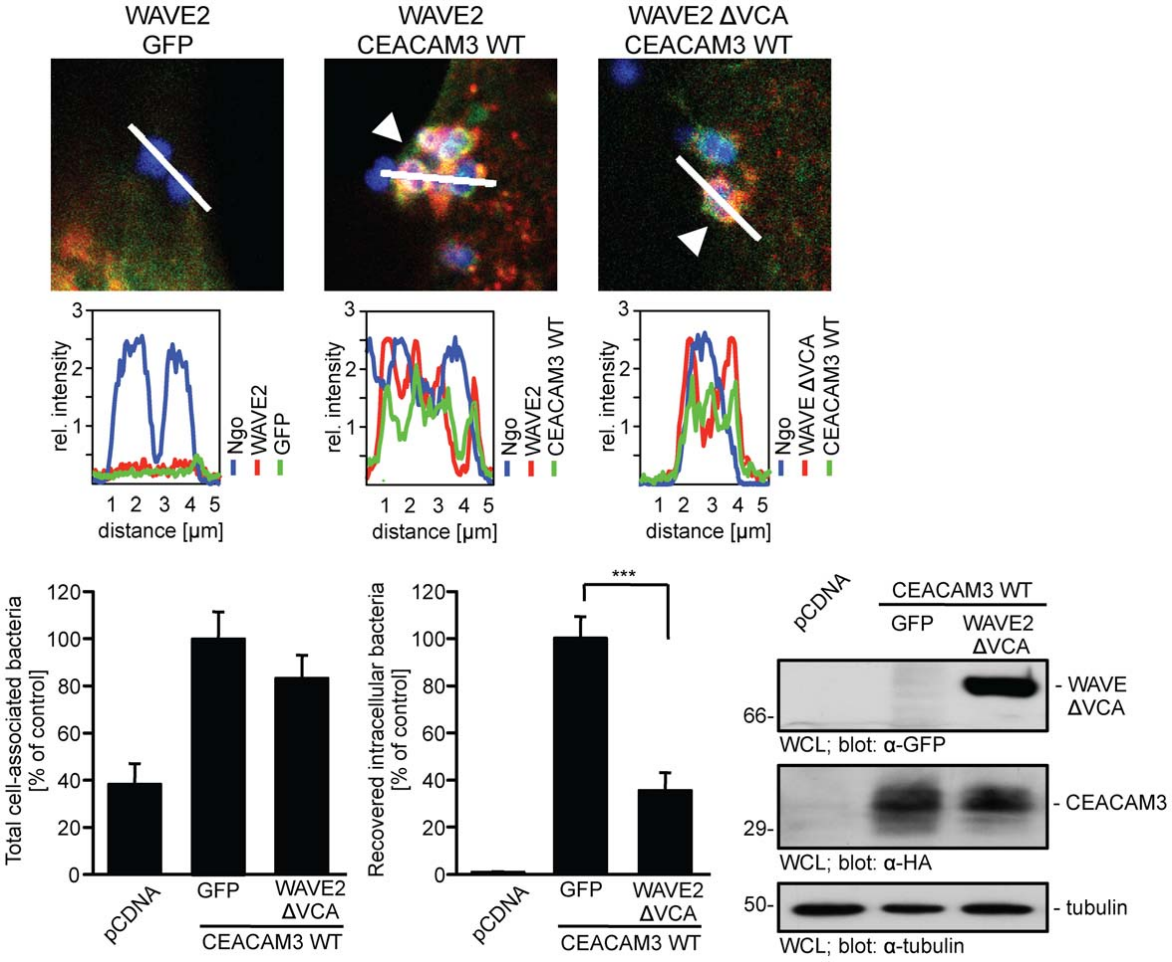
WAVE2 function is required during CEACAM3-mediated uptake of bacteria. (A) 293 cells were co-transfected with CEACAM3WT-GFP or GFP (green) together with myc-tagged WAVE2 or WAVE ΔVCA (red) and infected for 30 min with PacificBlue-labelled Opa_CEA_-expressing *N. gonorrhoeae* (blue). After fixation, WAVE2 was stained with anti-myc antibodies and samples were analysed by confocal laser scanning microscopy. Recruitment of WAVE2 or WAVE ΔVCA to cell-bound bacteria (arrowheads) in the presence of CEACAM3 was further visualized by line profiles representing the relative fluorescence intensity values in the three detection channels (lower panels). (B) 293 cells were transfected with pcDNA or a plasmid encoding HA-tagged CEACAM3 WT and co-transfected with either GFP alone or GFP-WAVE2 ΔVCA. Cells were infected for 30 minutes with Opa_CEA_-expressing *N. gonorrhoeae*. Parallel samples were analysed by bacterial adhesion assays (left panel) or gentamicin protection assays (middle panel). Bars represent mean values ± S.E.M of three independent experiments done in triplicate. Significance was tested using a paired, two-sided Student's t-test; ***, p<0.001. Expression of constructs was verified by Western blotting of whole cell lysates (WCL) with mAb against GFP, HA-tag, or tubulin as indicated (right panels).

Our finding of a Nck-mediated recruitment of the WAVE complex implicates that this activator of actin nucleation might be involved in actin cytoskeleton rearrangements during CEACAM3-mediated phagocytosis. To test the functional significance of the WAVE complex in bacterial uptake, we used a dominant negative variant of WAVE2 that lacks the carboxy-terminal VCA domain (WAVE2 ΔVCA). WAVE2 ΔVCA is not able to associate with the Arp2/3 complex and, therefore, disrupts WAVE-initiated actin nucleation [26]. Similar to wildtype WAVE2, WAVE2 ΔVCA was strongly recruited to sites of bacterial contact with CEACAM3 (Fig. 8A). This result suggests that the Arp2/3 complex-binding VCA domain of WAVE2 is not required for the Nck-mediated localization to phosphorylated CEACAM3. However, overexpression of WAVE2 ΔVCA severely impaired the CEACAM3-mediated uptake of *N. gonorrhoeae* without effecting bacterial binding to the cells (Fig. 8B). These results demonstrate that the Nck binding partner WAVE2 is involved in CEACAM3-mediated phagocytosis.

### The WAVE complex is involved in actin rearrangements during CEACAM3-mediated phagocytosis

**Figure 9:**
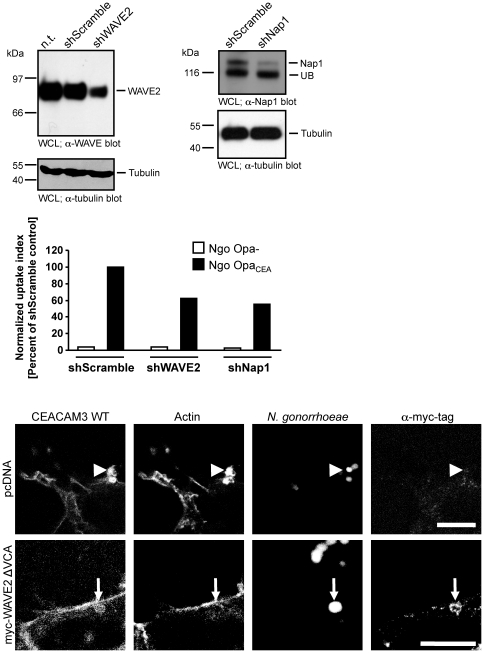
Knock-down of WAVE complex components interferes with CEACAM3-mediated uptake of bacteria. (A) 293 cells were transduced with lentiviral particles encoding shRNAs directed against WAVE2 or Nap1 or encoding a non-targeting shRNA (shScramble). Whole cell lysates (WCL) of the knock-down cells were analysed by Western blotting with antibodies against WAVE, Nap1 or tubulin. UB – unspecific band observed with the Nap1 antibody. (B) The indicated knock-down cells were infected with fluorescein-labelled non-opaque or Opa_CEA_-expressing gonococci for 1 h and internalization of bacteria was analysed by flow cytometry. Shown is a representative experiment repeated two times with similar results. (C) 293 cells were co-transfected with CEACAM3 WT-GFP together with myc-WAVE2 ΔVCA or the empty vector (pcDNA). Cells were infected for 30 min with PacificBlue-labelled Opa_CEA_-expressing *N. gonorrhoeae* (blue). After fixation, WAVE2 was stained with anti-myc antibodies and phalloidin-AlexaFluor546 was used to visualize f-actin. Samples were analysed by confocal laser scanning microscopy. In the absence of WAVE2 ΔVCA, CEACAM3-binding bacteria induce local accumulation of f-actin (arrowheads), whereas WAVE2 ΔVCA recruited to bacteria-engaged CEACAM3 suppresses local f-actin accumulation (small arrows). Bars indicate 10 µm.

To further corroborate the functional role of WAVE2 and the WAVE complex during CEACAM3-mediated uptake of bacteria, we generated cell populations with shRNA-mediated knock-down of WAVE2 or Nap1, the Nck-binding component of the WAVE complex. Furthermore, control cells were transduced with a recombinant lentivirus encoding a non-targeting, control shRNA (shScramble). After puromycin selection, the successful knock-down was verified by Western Blotting, which demonstrated that WAVE2 and Nap1 expression were reduced by about 70% and 60%, respectively (Fig. 9A). Virally transduced cell populations were transfected with a HA-tagged CEACAM3 construct. Upon infection with non-opaque *N. gonorrhoeae*, neither CEACAM3-transfected cell line internalized significant amounts of bacteria (Fig. 9B). However, Opa_CEA_ protein-expressing gonococci were efficiently internalized by the shScramble transduced cells. Strikingly, both the WAVE2- as well as the Nap1-knock-down cells showed a decreased uptake of Opa_CEA_ protein-expressing gonococci (Fig. 9B). Though the shRNA-mediated knock-down was incomplete, the 50 to 60% decrease of bacterial phagocytosis by the WAVE2- and Nap1-knock-down cells compared to the control cell (shScramble) corresponds well to the extent of diminished expression of these two proteins. These results corroborate the functional role of the WAVE complex in CEACAM3-initiated phagocytosis of bacteria.

During CEACAM3-initiated phagocytosis, GTP-loaded Rac stimulates massive actin polymerization leading to lamellipodial protrusions [15] and our results suggested that CEACAM3-associated Nck was also involved in this phenotype. Therefore, we speculated that the Nck-associated WAVE complex might be the critical Rac effector involved in actin polymerization in the vicinity of CEACAM3-binding bacteria. To address this question, we co-transfected 293 cells with CEACAM3 and either GFP or GFP-tagged, dominant-negative WAVE2 ΔVCA. Whereas a strong accumulation of f-actin was observed in GFP-expressing cells at the sites of bacteria-initiated CEACAM3 clustering, accumulation of f-actin was absent in WAVE2 ΔVCA expressing cells (Fig. 9C).

Together, these results suggest that Nck has a functional role in orchestrating CEACAM3-initiated signalling to the actin cytoskeleton by localizing the actin polymerization promoting activity of the WAVE complex. Accordingly, the adapter molecule Nck helps to convert the cytoplasmic domain of CEACAM3 into a sophisticated signalling hub, where upon receptor stimulation Rac GEFs such as Vav are brought into close proximity to Rac effectors such as WAVE to promote efficient, opsonin-independent phagocytosis of CEACAM3-attached microbes.

## Discussion

Several human specific pathogens are able to engage CEACAM family members to colonize mucosal surfaces [2], [4]). On the other hand, human granulocytes are equipped with CEACAM3 to detect and eliminate CEACAM-binding bacteria in an opsonin-independent manner [11], [15].

**Figure 10:**
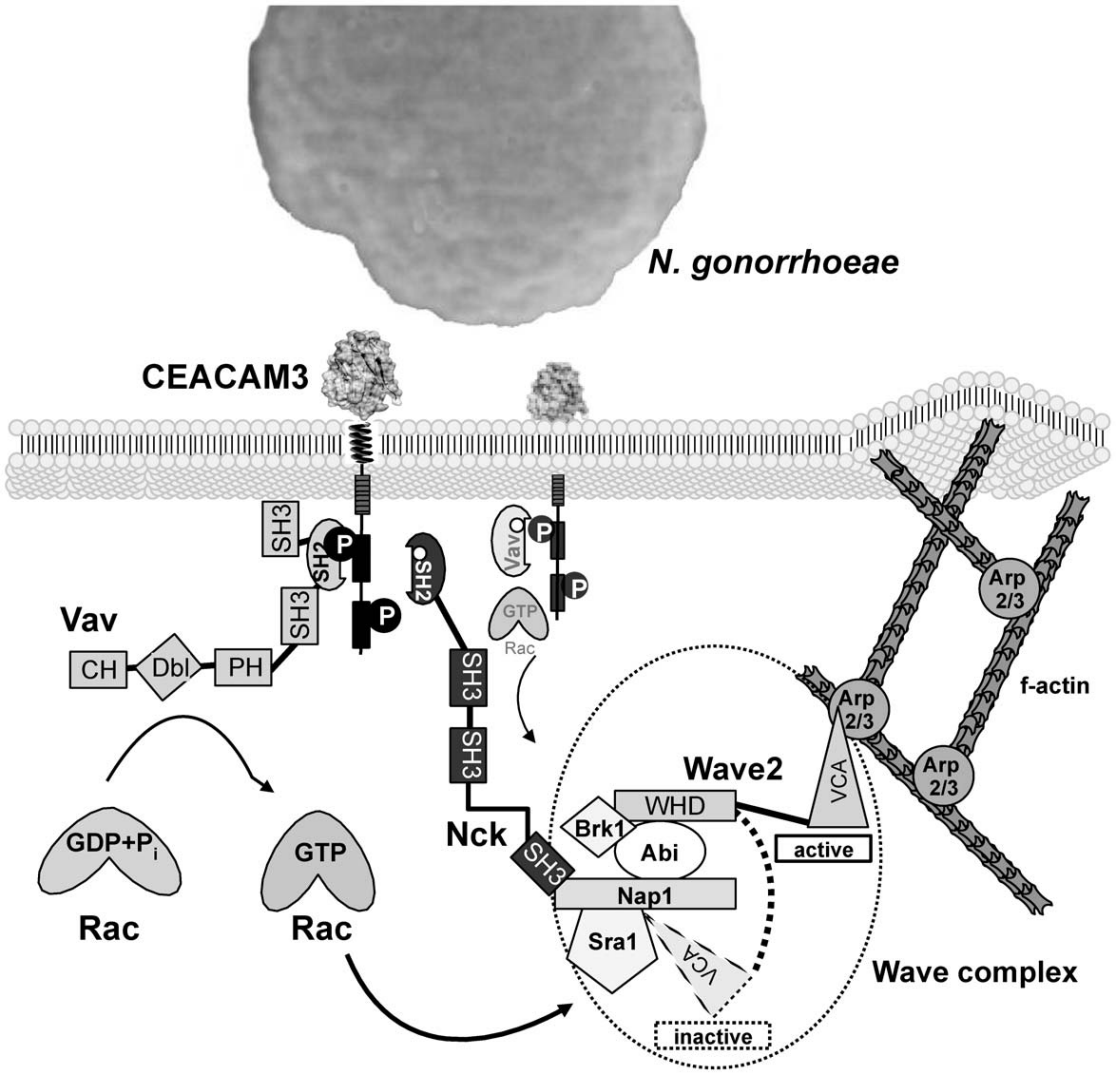
Current model of CECAM3 signal transduction. Upon engagement of CEACAM3 by CEACAM-binding pathogens (e.g. *Neisseria gonorrhoeae* expressing Opa_CEA_) the ITAM-like sequence in the cytoplasmatic domain of the receptor is phosphorylated by Src family kinases (SFKs) on two tyrosine residues (Y230 and Y241) that in turn serve as docking sites for several effector proteins. The Rac-GEF Vav directly binds to pY230 via its SH2 domain and activates Rac by facilitating GDP release. While Vav activates Rac, the adaptor molecule Nck is also recruited to CEACAM3 in a phosphotyrosine-dependent manner along with the WAVE complex. Nck constitutively associates with the WAVE complex, presumably by an SH3 domain-mediated interaction with Nap1. The CEACAM3-localized WAVE-complex can now be activated by a GTP-Rac induced conformational change from an inactive conformation (dotted wedge) to an active complex that exposes the VCA-domain of WAVE (solid wedge) to recruit the Arp2/3 complex. The activation of the Arp2/3 complex, which initiates nucleation of actin filaments, most likely mediates lamellipodia formation during the opsonin-independent phagocytosis of CEACAM3-binding bacteria.

Here we demonstrate that the adapter molecule Nck is a critical element in the CEACAM3-initiated signalling cascade that helps to connect bacterial recognition via CEACAM3 with rapid actin-dependent phagocytosis. Nck associates in a phosphotyrosine-dependent manner with the intracellular ITAM-like sequence of CEACAM3 and knock-down of Nck or overexpression of the isolated SH2 domain impair CEACAM3-mediated uptake of *N. gonorrhoeae*. Nck positions the WAVE complex to the clustered receptor promoting local actin polymerization and rapid engulfment of attached bacteria. These results suggest that the cytoplasmic domain of CEACAM3, by bringing together the Rac stimulator Vav as well as Rac effectors such as WAVE, functions as a specialized organizing center optimized to trigger efficient, opsonin-independent phagocytosis of CEACAM-binding bacteria (Fig. 10).

Nck adapter proteins have been recognized as important regulators of growth factor receptor- and integrin-stimulated signals that control the organization of the actin cytoskeleton [25]. For example, Nck adapter proteins are critical for the actin polymerization-driven dorsal ruffle formation upon growth factor stimulation, a process that resembles lamellipodia formation during phagocytosis [28]. Nck1 and Nck2 are co-expressed in most tissues and mediate redundant functions, as single knock-out of either gene does not impair the overall function of the organism, whereas deletion of both genes results in embryonic lethality [24]. In line with their overlapping function, Nck1 and Nck2 seem to interact with a similar set of proteins via either their SH2 or their SH3 domains [29]. Our studies also support the idea that Nck1 and Nck2 SH2 domains bind to the same target proteins, as both are able to precipitate tyrosine phosphorylated CEACAM3 in our assays. Crystallization of the Nck1 and Nck2 SH2 domains together with phosphorylated target peptides as well as biochemical binding studies have revealed that both adapters can accommodate a similar set of phospho-peptides [30]. The consensus motif for Nck-SH2 domain binding has revealed a preference for one or two acidic amino acids carboxy-terminal to the phospho-tyrosine in the form of pY-D/E-D/E-V [30]. Indeed, one of the two tyrosine residues within the ITAM-like sequence of CEACAM3 is followed by two negatively charged amino acids in the form of Y-E-E-L, which closely matches the characterized motif and supports the idea that the Nck SH2 domain directly associates with the cytoplasmic part of CEACAM3.

Importantly, Nck also participates in Fcγ receptor- and T-cell receptor (TCR)-initiated signaling events that involve ITAM phosphorylation [29], [31]. There, Nck associates with the activated receptors via additional, tyrosine-phosphorylated adapter molecules such as c-Cbl or SLP-76, respectively. In the context of T-cell stimulation, Nck is associated with the TCR complex via SLP-76 and recruits the Wiskott-Aldrich-Syndrome protein (WASP), which binds to the carboxy-terminal SH3 domain of Nck [32], [33]. Besides Nck, phosphorylated SLP-76 also binds the guanine nucleotide exchange factor Vav [34]. Interestingly, SLP-76 bears multiple Y-E-X-P motifs in its amino-terminal part that bind the SH2 domains of Nck and Vav, though they are clearly distinct from the optimal Nck SH2 (pY-D/E-D/E-V) or Vav SH2 (pY-M-E-P) recognition sequences as determined by phospho-peptide scanning [30], [35]. A similar situation exists for CEACAM3, where the Y-E-E-L motif surrounding Y-230 directly associates with the Vav SH2 domain [22] and might also be responsible for Nck binding.

By the help of Nck, SLP-76 brings into proximity a stimulator (Vav) as well as an effector (WASP) of GTP-bound small Rho GTPases to locally organize the actin cytoskeleton [33], [34]. It is enticing to speculate that a similar role is carried out by the cytoplasmic domain of CEACAM3 and thereby this receptor directly, without intermediary adaptor proteins, recruits the machinery for rapid actin polymerization. The assembly of such an actin regulating signalling complex by the cytplasmic portion of CEACAM3 might also explain why the ITAM-like sequence of this receptor can trigger efficient phagocytosis in the absence of Syk or Zap70 [36]. These two tyrosine kinases are essential for cytoskeletal rearrangements initiated by canonical ITAM motifs [13], [37]. For example, inhibition of Syk activity in macrophages compromises FcγR-mediated phagocytosis. In contrast, CEACAM3-mediated uptake of bacteria is not influenced by Syk inhibitors in primary granulocytes or by co-expression of Syk in CEACAM3-transfected cell lines ([36]; Pils and Hauck, unpublished observations). Together, these findings suggest that CEACAM3 short wires receptor stimulation with efficient phagocytosis and, similar to phosphorylated SLP-76, achieves rapid reorganization of the actin cytoskeleton by optimizing the local environment for Rho GTPase signaling.

Despite the conceptual similarities, there is one important difference in the SLP-76-mediated coordination of TCR-induced actin rearrangements at the immunological synapse and CEACAM3-induced actin polymerization during phagocytosis. Upon TCR stimulation, the small GTPase Cdc42 is thought to trigger WASP-mediated actin cytoskeleton rearrangements [33], [38]. In contrast, CEACAM stimulation in granulocytes is tightly linked to Rac GTP loading, and dominant-negative versions of Cdc42 do not interfere with *N. gonorrhoeae* internalization in CEACAM3-transfected human cell lines or primary human granulocytes [15], [23].

How the selective recruitment and stimulation of either Cdc42 or Rac is achieved is currently unknown, as both TCR- as well as CEACAM3-initiated signalling involve the guanine nucleotide exchange factor Vav [22], [39], [40]. Nevertheless, our findings that Nck associates with phosphorylated CEACAM3 and recruits the WAVE2 complex shed light on the downstream events following Rac GTP loading. WAVE2 is part of a pentameric complex that is intrinsically inactive [41], [42]. Besides WAVE2, Abi, and Brk1, the complex contains the proteins Sra and Nap1, which seem to shield the carboxy-terminal VCA domain of WAVE2, thereby preventing WAVE-initiated Arp2/3 complex stimulation and actin nucleation [27], [43]. Though the precise details are currently unknown, the actin nucleation promoting activity of the WAVE complex appears to depend on multiple inputs including association with GTP-loaded Rac, binding to phosphoinositides, and phosphorylation of WAVE [42], [44], [45] that all seem to alter the conformation of the complex (Fig. 10).

Interestingly, one integral component of the WAVE complex, Nap1 (also termed NCKAP1 or Hem2), has been initially identified as an Nck binding partner in a yeast-two-hybrid screen [46], [47]. Interaction between Nap1 and Nck is mediated by proline-rich sequences in Nap1 and Nck SH3 domains allowing a constitutive, phosphorylation-independent association as also detected in our analysis. Though direct binding of the SH3 domain of IRSp53, a BAR-domain containing protein localized at lamellipodial membrane protrusions, has been shown to contribute to activation of the WAVE complex, it is not known, if the SH3 domain-mediated association of Nck with Nap1 can result in an allosteric activation of the complex [27]. However, the Nck-Nap1 interaction could clearly contribute to the subcellular localization of the WAVE complex, thereby directing the actin nucleation machinery to clustered and tyrosine phosphorylated CEACAM3. Moreover, Nck could affect WAVE complex activation indirectly. In particular, Nck is known to bind the cytoplasmic tyrosine kinase Abl, that phosphorylates WAVE2 at tyrosine residue Y150 providing an essential post-translational modification for full activation of the WAVE complex [45], [48]. Abl directly binds to Abi, another constituent of the WAVE complex, and additional interactions between Nck SH3 domains and Abl might facilitate or stabilize such an association.

Nck also associates with the actin nucleation promoting factor N-WASP, an effector of GTP-loaded Cdc42 [25]. Nck/N-WASP driven actin polymerization is critical for pedestal formation in mammalian cells infected with enteropathogenic *E. coli* and for actin-based intracellular motility driven by the vaccinia virus protein A36R [49]–[51]. Based on our data, we can not rule out an involvement of WASP family members in CEACAM3-induced lamellipodia formation. However, the dependence on Rac-GTP instead of Cdc42-GTP point to the WAVE complex instead of WASP as potential downstream elements. In addition, other cellular factors, such as Spire or formins, could contribute to the proper organization of the actin cytoskeleton during the complex process of CEACAM3-mediated internalization, either in parallel or in a sequential manner. Indeed, even in the absence of a functional ITAM-like sequence or upon deletion of the complete cytoplasmic domain, an inefficient, residual uptake of bacteria via CEACAM3 has been observed [15], [16], which might also explain the remaining uptake observed in Nck1/Nck2-deficient cells. However, based on our CLEM results on the lack of lamellipodial protrusions in Nck1/2-deficient cells and based on the severe reduction of actin polymerization in the vicinity of clustered CEACAM3 in WAVE2 ΔVCA-expressing cells we suggest that the Nck-mediated local enrichment of the WAVE complex is the major actin nucleation promoting activity during CEACAM3-mediated phagocytosis of bacteria.

Our work reveals the extremely compact organization of CEACAM3-initiated signalling that connects the opsonin-independent recognition of CEACAM-binding pathogens via the extracellular immunoglobulin domain with rapid phagocytosis initiated by the cytoplasmic domain. By recruiting in a tyrosine-phosphorylation dependent manner the guanine nucleotide exchange factor Vav as well as the Nck-WAVE module, the CEACAM3 ITAM-like sequence elegantly integrates an upstream activator and a downstream effector to provide an optimized subcellular microenvironment for Rac-dependent actin rearrangements. Indeed, CEACAM3-mediated uptake by human granulocytes is exceptionally fast and results in a large number of internalized bacteria within 15 minutes after phagocyte encounter [15], [18], [22]. The involvement of few, but ubiquitously expressed signalling components not only explains why CEACAM3 can function as an efficient phagocytic receptor in different transfected cell lines, but also points to a minimal set of proteins needed for rapid and efficient engulfment of particles by human granulocytes.

## References

[1] Hauck CR, Agerer F, Muenzner P, Schmitter T (2006). Cellular adhesion molecules as targets for bacterial infection.. Eur J Cell Biol.

[2] Virji M (2009). Pathogenic *Neisseriae*: surface modulation, pathogenesis and infection control.. Nat Rev Microbiol.

[3] Hammarstrom S (1999). The carcinoembryonic antigen (CEA) family: structures, suggested functions and expression in normal and malignant tissues.. Semin Cancer Biol.

[4] Muenzner P, Bachmann V, Hentschel J, Zimmermann W, Hauck CR (2010). Human-specific bacterial pathogens block shedding of epithelial cells by stimulating integrin activation.. Science.

[5] Muenzner P, Rohde M, Kneitz S, Hauck CR (2005). CEACAM engagement by human pathogens enhances cell adhesion and counteracts bacteria-induced detachment of epithelial cells.. J Cell Biol.

[6] Chen T, Zimmermann W, Parker J, Chen I, Maeda A (2001). Biliary glycoprotein (BGPa, CD66a, CEACAM1) mediates inhibitory signals.. J Leukocyte Biol.

[7] Pantelic M, Kim YJ, Bolland S, Chen I, Shively J (2005). Neisseria gonorrhoeae kills carcinoembryonic antigen-related cellular adhesion molecule 1 (CD66a)-expressing human B cells and inhibits antibody production.. Infect Immun.

[8] Boulton IC, Gray-Owen SD (2002). Neisserial binding to CEACAM1 arrests the activation and proliferation of CD4+ T lymphocytes.. Nat Immunol.

[9] Youssef AR, van der Flier M, Estevao S, Hartwig NG, van der Ley P (2009). Opa+ and Opa- isolates of Neisseria meningitidis and Neisseria gonorrhoeae induce sustained proliferative responses in human CD4+ T cells.. Infect Immun.

[10] Gray-Owen SD, Blumberg RS (2006). CEACAM1: contact-dependent control of immunity.. Nat Rev Immunol.

[11] Pils S, Gerrard D, Meyer A, Hauck. CR (2008). CEACAM3: an innate immune receptor directed against human-resticted bacterial pathogens.. Intl J Med Microbiol.

[12] Abram CL, Lowell CA (2007). The expanding role for ITAM-based signaling pathways in immune cells.. Sci STKE.

[13] Greenberg S (1995). Signal transduction of phagocytosis.. Trends Cell Biol.

[14] Booth JW, Telio D, Liao EH, McCaw SE, Matsuo T (2003). Phosphatidylinositol 3-kinases in carcinoembryonic antigen-related cellular adhesion molecule-mediated internalization of *Neisseria gonorrhoeae*.. J Biol Chem.

[15] Schmitter T, Agerer F, Peterson L, Muenzner P, Hauck CR (2004). Granulocyte CEACAM3 is a phagocytic receptor of the innate immune system that mediates recognition and elimination of human-specific pathogens.. J Exp Med.

[16] Billker O, Popp A, Brinkmann V, Wenig G, Schneider J (2002). Distinct mechanisms of internalization of *Neisseria gonorrhoeae* by members of the CEACAM receptor family involving Rac1- and Cdc42- dependent and -independent pathways.. EMBO J.

[17] McCaw SE, Liao EH, Gray-Owen SD (2004). Engulfment of *Neisseria gonorrhoeae*: revealing distinct processes of bacterial entry by individual carcinoembryonic antigen-related cellular adhesion molecule family receptors.. Infect Immun.

[18] Schmitter T, Pils S, Weibel S, Agerer F, Buntru A (2007). Opa proteins of pathogenic *Neisseriae* initiate Src-kinase-dependent or lipid raft-mediated uptake via distinct human CEACAM isoforms.. Infect Immun.

[19] Muenzner P, Bachmann V, Kuespert K, Hauck CR (2008). The CEACAM1 transmembrane domain, but not the cytoplasmic domain, directs internalization of human pathogens via membrane-microdomains.. Cell Microbiol.

[20] McCaw SE, Schneider J, Liao EH, Zimmermann W, Gray-Owen SD (2003). Immunoreceptor tyrosine-based activation motif phosphorylation during engulfment of *Neisseria gonorrhoeae* by the neutrophil-restricted CEACAM3 (CD66d) receptor.. Mol Microbiol.

[21] Buntru A, Zimmermann T, Hauck CR (2009). FRET-based subcellular visualization of pathogen-induced host receptor signalling.. BMC Biol.

[22] Schmitter T, Pils S, Sakk V, Frank R, Fischer KD (2007). The granulocyte receptor CEACAM3 directly associates with Vav to promote phagocytosis of human pathogens.. J Immunol.

[23] Hauck CR, Meyer TF, Lang F, Gulbins E (1998). CD66-mediated phagocytosis of Opa_52_
*Neisseria gonorrhoeae* requires a Src-like tyrosine kinase- and Rac1-dependent signalling pathway.. EMBO J.

[24] Bladt F, Aippersbach E, Gelkop S, Strasser GA, Nash P (2003). The murine Nck SH2/SH3 adaptors are important for the development of mesoderm-derived embryonic structures and for regulating the cellular actin network.. Mol Cell Biol.

[25] Buday L, Wunderlich L, Tamas P (2002). The Nck family of adapter proteins: regulators of actin cytoskeleton.. Cell Signal.

[26] Miki H, Suetsugu S, Takenawa T (1998). WAVE, a novel WASP-family protein involved in actin reorganization induced by Rac.. EMBO J.

[27] Derivery E, Gautreau A (2010). Generation of branched actin networks: assembly and regulation of the N-WASP and WAVE molecular machines.. Bioessays.

[28] Ruusala A, Pawson T, Heldin CH, Aspenstrom P (2008). Nck adapters are involved in the formation of dorsal ruffles, cell migration, and Rho signaling downstream of the platelet-derived growth factor beta receptor.. J Biol Chem.

[29] Lettau M, Pieper J, Janssen O (2009). Nck adapter proteins: functional versatility in T cells.. Cell Commun Signal.

[30] Frese S, Schubert WD, Findeis AC, Marquardt T, Roske YS (2006). The phosphotyrosine peptide binding specificity of Nck1 and Nck2 Src homology 2 domains.. J Biol Chem.

[31] Izadi KD, Erdreich-Epstein A, Liu Y, Durden DL (1998). Characterization of Cbl-Nck and Nck-Pak1 interactions in myeloid FcgammaRII signaling.. Exp Cell Res.

[32] Rivero-Lezcano OM, Marcilla A, Sameshima JH, Robbins KC (1995). Wiskott-Aldrich syndrome protein physically associates with Nck through Src homology 3 domains.. Mol Cell Biol.

[33] Zeng R, Cannon JL, Abraham RT, Way M, Billadeau DD (2003). SLP-76 coordinates Nck-dependent Wiskott-Aldrich syndrome protein recruitment with Vav-1/Cdc42-dependent Wiskott-Aldrich syndrome protein activation at the T cell-APC contact site.. J Immunol.

[34] Bubeck Wardenburg J, Pappu R, Bu JY, Mayer B, Chernoff J (1998). Regulation of PAK activation and the T cell cytoskeleton by the linker protein SLP-76.. Immunity.

[35] Songyang Z, Shoelson SE, McGlade J, Olivier P, Pawson T (1994). Specific motifs recognized by the SH2 domains of Csk, 3BP2, fps/fes, GRB-2, HCP, SHC, Syk, and Vav.. Mol Cell Biol.

[36] Sarantis H, Gray-Owen SD (2007). The specific innate immune receptor CEACAM3 triggers neutrophil bactericidal activities via a Syk kinase-dependent pathway.. Cell Microbiol.

[37] Crowley MT, Costello PS, Fitzer-Attas CJ, Turner M, Meng F (1997). A critical role for Syk in signal transduction and phagocytosis mediated by Fcgamma receptors on macrophages.. J Exp Med.

[38] Stowers L, Yelon D, Berg LJ, Chant J (1995). Regulation of the polarization of T cells toward antigen-presenting cells by Ras-related GTPase CDC42.. Proc Natl Acad Sci U S A.

[39] Fischer KD, Zmuldzinas A, Gardner S, Barbacid M, Bernstein A (1995). Defective T-cell receptor signalling and positive selection of Vav-deficient CD4+ CD8+ thymocytes.. Nature.

[40] Zhang R, Alt FW, Davidson L, Orkin SH, Swat W (1995). Defective signalling through the T- and B-cell antigen receptors in lymphoid cells lacking the vav proto-oncogene.. Nature.

[41] Derivery E, Lombard B, Loew D, Gautreau A (2009). The Wave complex is intrinsically inactive.. Cell Motil Cytoskeleton.

[42] Lebensohn AM, Kirschner MW (2009). Activation of the WAVE complex by coincident signals controls actin assembly.. Mol Cell.

[43] Takenawa T, Suetsugu S (2007). The WASP-WAVE protein network: connecting the membrane to the cytoskeleton.. Nat Rev Mol Cell Biol.

[44] Danson CM, Pocha SM, Bloomberg GB, Cory GO (2007). Phosphorylation of WAVE2 by MAP kinases regulates persistent cell migration and polarity.. J Cell Sci.

[45] Leng Y, Zhang J, Badour K, Arpaia E, Freeman S (2005). Abelson-interactor-1 promotes WAVE2 membrane translocation and Abelson-mediated tyrosine phosphorylation required for WAVE2 activation.. Proc Natl Acad Sci U S A.

[46] Kitamura T, Kitamura Y, Yonezawa K, Totty NF, Gout I (1996). Molecular cloning of p125Nap1, a protein that associates with an SH3 domain of Nck.. Biochem Biophys Res Commun.

[47] Kitamura Y, Kitamura T, Sakaue H, Maeda T, Ueno H (1997). Interaction of Nck-associated protein 1 with activated GTP-binding protein Rac.. Biochem J.

[48] Stuart JR, Gonzalez FH, Kawai H, Yuan ZM (2006). c-Abl interacts with the WAVE2 signaling complex to induce membrane ruffling and cell spreading.. J Biol Chem.

[49] Frischknecht F, Moreau V, Rottger S, Gonfloni S, Reckmann I (1999). Actin-based motility of vaccinia virus mimics receptor tyrosine kinase signalling.. Nature.

[50] Gruenheid S, DeVinney R, Bladt F, Goosney D, Gelkop S (2001). Enteropathogenic E. coli Tir binds Nck to initiate actin pedestal formation in host cells.. Nat Cell Biol.

[51] Hayward RD, Leong JM, Koronakis V, Campellone KG (2006). Exploiting pathogenic Escherichia coli to model transmembrane receptor signalling.. Nat Rev Microbiol.

